# Atrous Convolutions and Residual GRU Based Architecture for Matching Power Demand with Supply

**DOI:** 10.3390/s21217191

**Published:** 2021-10-29

**Authors:** Samee Ullah Khan, Ijaz Ul Haq, Zulfiqar Ahmad Khan, Noman Khan, Mi Young Lee, Sung Wook Baik

**Affiliations:** Sejong University, Seoul 143-747, Korea; samee@sju.ac.kr (S.U.K.); hijaz3797@gmail.com (I.U.H.); mzulfiqar3797@gmail.com (Z.A.K.); nomank3797@gmail.com (N.K.); miylee@sejong.ac.kr (M.Y.L.)

**Keywords:** deep learning, energy management system, energy consumption, renewable energy, smart grid

## Abstract

Nowadays, for efficient energy management, local demand-supply matching in power grid is emerging research domain. However, energy demand is increasing day by day in many countries due to rapid growth of the population and most of their work being reliant on electronic devices. This problem has highlighted the significance of effectively matching power demand with supply for optimal energy management. To resolve this issue, we present an intelligent deep learning framework that integrates Atrous Convolutional Layers (ACL) with Residual Gated Recurrent Units (RGRU) to establish balance between the demand and supply. Moreover, it accurately predicts short-term energy and delivers a systematic method of communication between consumers and energy distributors as well. To cope with the varying nature of electricity data, first data acquisition step is performed where data are collected from various sources such as smart meters and solar plants. In the second step a pre-processing method is applied on raw data to normalize and clean the data. Next, the refined data are passed to ACL for spatial feature extraction. Finally, a sequential learning model RGRU is used that learns from complicated patterns for the final output. The proposed model obtains the smallest values of Mean Square Error (MSE) including 0.1753, 0.0001, 0.0177 over IHEPC, KCB, and Solar datasets, respectively, which manifests better performance as compared to existing approaches.

## 1. Introduction

**Figure 1:**
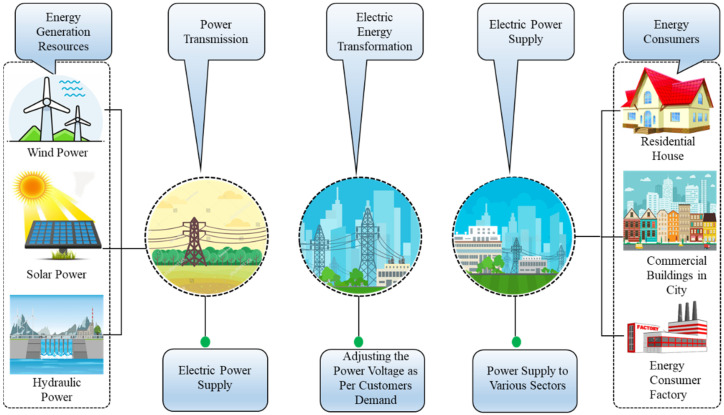
The overall mechanism of the energy generation, transformation, and consumption.

In smart grids energy management through automated techniques for future energy generation and consumption prediction is a fascinating field of research. Smart grids are safe and authentic venues that efficiently transmit energy among various consumers. The entire process of electric energy from generation resources (wind, solar, hydro) to consumption (residential, commercial, industries) [1,2] is shown in Figure 1. The energy consumption at the consumer side completely affects the energy generation at power plants. Most of the consumers are inexpert and have no knowledge of how to demand energy from power grids, resulting in a waste of energy and financial loss. In the meantime, the suppliers at the production unit intend to reduce the total expense as much as possible to get a balanced level of energy generation which needs proper timing and management schemes. For better planning, an efficient model is highly required that can precisely predict energy consumed in houses/industries and generated at power plants. A smart grid is responsible for the stable state of energy between generation and consumption [3].

Electricity is a basic ingredient of human life. It is necessary to keep the balance between the supplier in the grid and real consumption demand to take full advantage of its usage. Due to increasing demand of electrical devices such as electric cars, microwave ovens, air purifiers, networking devices etc., the management of power grids faces numerous challenges. It is estimated that in 2050, 62% of energy will be provide by renewable sources. Moreover, the quantity of green energy created in 2019 is 27% globally while the capacity of the total production in Slovakia is only 13%. However, it is very difficult to connect the renewable energy sources with smart grids because it entirely relies on weather conditions. From this perspective, renewable energy sources are unreliable without a sophisticated management system. To address such problems, energy storage device (i.e., battery) is the best solution that preserve unused energy for later usage. Additionally, Artificial Intelligence (AI)-based battery health prediction models significantly improve the companies’ planning through which energy management teams generate the energy based on user’s demand [4]. Secondly, the temperature within electric wires rises when electricity is transmitted over long distances, resulting in considerable energy loss in the form of heat. At the end, these electricity losses are compensated from consumers. For such type of problem, decentralization method is better to use where large energy plants transmitted the energy to the local smart grid which have sufficient knowledge about the demand of consumers. Moreover, to establish efficient energy transmission the proposed model is suitable for matching demand with power supply. Another problem is hardware failure in electrical devices like electric cars where a significant amount of time is required for charging. For fast charging, supercharges consumes huge amount of energy, resulting high energy demand from smart grid. Therefore, to speed up this procedure a stable energy prediction model is necessary that can meet the desires of the consumers on time.

There are two main issues arising in energy generation, including sustainable development and the worldwide weather change [5]. Due to the increasing number of the population the consumption of energy is rising by 2% every year, where energy generation is totally depending on fossil fuels including coal, oil, and natural gas [6]. Fossil fuels is the major resource of energy generation which has been widely used throughout the years. The usage of fossil fuels in huge amounts instigated their lack and also generated some environmental problems that affect the living health and disturb the global climate [7]. Furthermore, the development of fossil fuels takes many years, whereas the current energy generated procedures are quicker than fossil fuels. As a result, the energy generation industries are presenting interest in renewable energy sources [8]. Renewable energy sources are considered the best substitutes for fossil fuels because they are naturally created on geographical area [9]. However, their usage with unpredictability that compromises the stability and dependability of large-scale renewable energy facilities [5]. Energy prediction facilities at renewable energy is a critical component of future stabilization and development [10]. Accurate energy consumption and generation prediction is a challenging task due to the unreliable, unexpected, and inconsistent nature of energy data. Regarding this, various resource energy forecasting has been examined in recent years to address the problems which have mainly arisen due to the excessive number of renewable energy resources and power plants globally [11]. The literature has described several approaches for short- and long-term energy consumption and generation. The majority of future renewable energy prediction approaches are based on physical models that estimate and predict energy using weather and power station data [12]. Most of the physical approaches are inadequate and unsuitable for short-term energy predictions [6]. In the literature, several statistical techniques including Hammerstein model, autoregressive moving average, Bayesian-based adaptive model, Kalman filter, and Markov chain model are commonly used for both energy consumption and generation [13]. The statistical techniques generate precise predictions; unfortunately, majority of them are unable to manage long-term energy predictions due to being linear in nature [6].

Machine Learning (ML) and Deep Learning (DL) have proved to be useful techniques for energy generation prediction [11] and intelligent communication [14]. Many AI-based methods have been proposed to improve renewable energy [15]. Numerous time horizons have been studied to estimate renewable energy generation including minutely, hourly, daily, weekly, and monthly, depending on the goals of the forecasting [16]. For the renewable energy prediction, ML-based techniques are commonly used including k-nearest neighbors, Support Vector Regression (SVR), random forest, multiple linear regression, support vector machines, and gradient boosting among others. Despite this, few well known sequential learning models such as long short-term memory and gated recurrent units are widely used for energy generation that give satisfactory results [17].

Due to the significant role and wider application of renewable energy, a considerable body of literature on the prediction of renewable energy has been published [18]. However, ML approaches faced numerous challenges because they can only work on a fixed length of data. Similarly, deep learning models, particularly CNNs, extract less meaningful information from time series data. Few ML- and DL-based models revealed incredible performance in real-time energy consumption and generation prediction, especially in dynamic environmental conditions [19]. Therefore, this study aimed to use a hybrid DL approach based on ACL and RGRU to enhance short-term horizons for both energy consumption and generation predictions. Our novel hybrid framework is beneficial for efficient energy management systems. The proposed network extracts discriminative and representative features from sequential data via ACL and forwards it to the RGRU that learns spatial patterns from sequence. Our main contributions in this study are as follows:

1. Sometimes, smart sensors generate unusual data due to several reasons such as metering fault, weather condition, irregular customer consumption etc., which requires data pre-processing strategies to refine the dataset before the training process. Therefore, in this work a pre-processing step is applied to remove abnormalities from raw input data including outlier reduction, filling missing values etc.
2. Conventional approaches followed a single model for energy consumption and generation prediction, which are unable to efficiently extract spatiotemporal patterns and also generate false predictions with high error rates. Therefore, we proposed a hybrid network with the integration of ACL and RGRU that minimize error score in generation as well as consumption.
3. Matching consumption with production becomes a more acute challenge in a sustainable energy system with increasing amounts of renewable energy that offer fluctuations and unpredictability. To overcome this problem, a generalized hybrid model is developed that efficiently predicts both generation and consumption energy.
4. The reliability of the model is assessed over Mean Square Error (MSE), Root Mean Square Error (RMSE), and Mean Absolute Error (MAE). The efficiency of the proposed model is evaluated over benchmark datasets which shows that the proposed framework greatly reduces the error rate as compared to other existing models.

The remaining paper is arranged as follows: Section 2 briefly describes the literature study about energy prediction methods. A brief description about the proposed framework is delivered in Section 3. The experimental results and performance assessment details are provided in Section 4, while Section 5 is directed towards the overall research conclusion with limitation and future research guidelines.

## 2. Literature Review

Energy production is a fundamental industrial sector where its preservation is one of the most essential aspects that enhances the industrial energy efficiency. A proper energy management is necessary for every company. If the energy efficiency does not improve, sustainable development will still produce a significant amount of energy.

The worldwide renewable energy industry had overall revenues of $692.8 billion in the year 2020, reflecting an 8.9% Compound Annual Growth Rate (CAGR) from 2016 to 2020 [20]. There are numerous companies which are responsible for the generation, transmission, and supply of electricity. In South Korea, the largest electric utility is Korea Electric Power Corporation (KEPCO). This company engages in business to obtain legitimate goals, which include electric power assets innovation, production, distribution, transformation and transmission of electric power, as well as marketing, research, technological advancement etc. Korea Western Power (KWP) is another company that generates and transmits the electrical energy to residential, commercial, and industrial sectors. Furthermore, it also maintains heat in power stations, and power storage plants. Another generation company known as Korea Hydro and Nuclear Power (KHNP) controlled nuclear power plants that produces natural gas, water, and electric energy. POSCO energy is involved in power production, battery storage, and renewable energy. For almost 50 years, POSCO energy has provided reliable energy to the metropolitan regions.

Energy for industries is one of the most powerful and profitable sources in the contemporary economy. However, most energy firms are unaware of the energy generation potential and do not use cutting-edge technology to improve their procedures. The world is faced with serious energy issues and installed smart machines in industry require significant amounts of energy to operate. Energy companies urgently need to enhance their forecast analysis tools to save costs, conserve energy, and deliver better service to customers. To accomplish this job, AI-based model is the best choice in the industry sectors for the purpose of predictive analytics. The energy forecasting literature can be divided into two major categories: energy consumption and generation approaches.

### 2.1. Energy Consumtpion Prediction Approaches

Machine learning-based models are commonly used in various domains such as heating load prediction [21], electrical energy generation and consumption prediction which are comparatively old literature [22], including Linear and multiple regression [23,24,25], clustering [26], SVR [27], Extreme Learning Machine (ELM) [28] among others are the main methods. The forecasting method that receives the most attention is Short-Term Load Forecasting (STLF). For example, Ceperic et al. proposed that the SVR technique used for a short period of power demands prediction [29]. Furthermore, they presented two major significant enhancements over the present forecasting methods based on SVR. The first improvement is the process inputs followed by the feature selection algorithms. They also implemented particle swarm global optimization to enhance the hyperparameters of SVR. Finally, the system was tested over two power predicting datasets and compare results with the existing approaches. In later study for STLF, Li et al. [28] predicted energy using evolutionary ELM and wavelet transform. To lead the ELM in selecting the optimal parameters from the provided input weights, the artificial bee colony algorithm is used. Based on public utility data, the authors accomplished substantial findings from various parts of North America and ISO New England. To compute the consumers demand feedback, Chen et al. [30] proposed a short-term forecasting model based on SVR for office buildings. In another approach, Wu et al. [31] developed a gradient boosting framework for short-term prediction using multiple kernel which delivers further flexibility as compared to conventional kernel approaches.

Due to the increase in popularity of DL, scientists mostly used it for energy load prediction [21,32] for better performance. Literature studies related to energy prediction based on DL approaches show incredible performance [33]. For instance, Kong et al. [34] proposed STLF LSTM which mainly focus on handling the power consumed by residential areas which leads the accurate forecasting results. Another study [35] developed a hybrid approach for energy prediction of domestic buildings in which genetic algorithms and DL were combined with LSTM to suggest the best objective function along with hidden neurons for the prediction of energy. This approach is checked across the data from commercial and residential households for VSTLF forecasting, and the results are better than those of the traditional forecasting models. Wu et al. [31] used the transfer regression method based on multiple kernel learning to predict the energy and perform experiments over the data from residential houses to demonstrate the significant boundaries of reduced error rate. Likewise, the latest studies employed a deep autoencoder to convert the low to high level features representation and at the end they applied a cluster algorithm which is known as an adoptive self-organizing map [36].

### 2.2. Energy Generation Prediction Approaches

In recent decades, experts have changed their attention to renewable energy sources for predicting energy generation. These energy sources have been extensively used for power generation because of their simplicity and renewable nature. The main problem in electricity generation from renewable sources is ensuring its long-term reliability. Energy generated through different resources mainly depend on various environmental factors including weather and wind (speed and direction). These uncontrollable factors make the prediction issue more difficult. Numerous types of methods have been used for energy prediction generated through wind, hydropower, and solar. As in this study, we utilized the solar dataset, therefore related literature is covered.

Solar energy is an infinite renewable energy source which does not release any gases or carbon because it does not use fuel and considered as one of the favorable technologies for energy generation. In this technology, sunlight radiation plays a significant role with varying time scales. Several ML and DL-based models have been proposed for proper management and prediction in a low computational manner [37]. Regarding this, Aslam et al. [38] investigated numerous DL methods including RNN, GRU, LSTM, SVR, and FFNN for short-term solar radiation prediction. Torres-Barran et al. [39] explored regression models such as random forest, gradient boost, and XGBoost for the prediction of energy generated through solar. Next, Saloux et al. [40] applied decision tree, SVM, and artificial neural network for solar plant heat prediction, whereas Sun et al. [41] developed a CNN-based prediction method of photovoltaics energy. Similarly, Torres et al. [42] introduced a feed forward neural network to forecast the power generated via PV. Few researchers used ML algorithms for solar energy generation prediction, for instance, Liu et al. [43] investigated SVM and regression-based approaches while ALKandari et al. [44] explored both statistical and ML methods for future solar energy prediction.

## 3. The Proposed Framework

**Figure 2:**
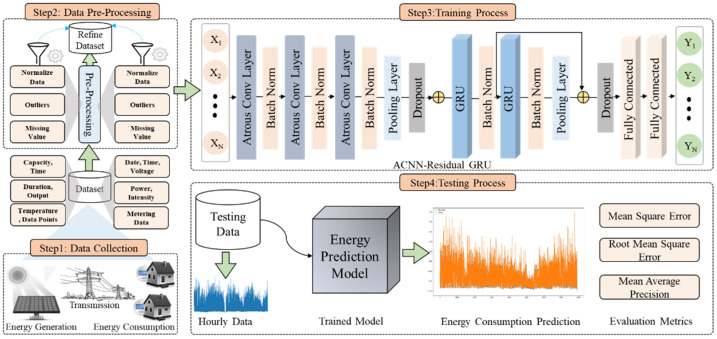
The proposed framework for short-term electricity generation and consumption prediction.

A smart grid is a venue that is responsible for managing and supplying energy among consumers with diverse levels of energy expenditure. A power grid along with a proper supervision system accomplishes the demand of consumers in an efficient way and saves massive amounts of energy for the future. In contrast, conventional grids willingly distribute energy as per consumers’ demand, without any knowledge about the consumer behaviors in case of erroneous usage and environment conditions, among others, that yield an incorrect consumption of energy. Meanwhile, a smart grid preserves and tracks the energy demands-related information individually. However, in most of the situation grids demonstrate weak performance due to work overload, or they do not have consumers’ energy demand data. Therefore, an automatic system is highly desired that accurately predicts the energy, management between consumer and suppliers, and removes anomalies from the commercial and residential sectors’ energy data. Concerning this, we propose a novel framework that precisely predicts the consumed and renewable energy that assists the smart grid during the transmission of energy on user demands. The complete scenario of the proposed framework is shown in Figure 2 where significant steps are briefly explained in the subsequent sections. The model learning and energy consumption prediction procedure is given in Algorithms 1 and 2.
**Algorithm 1** Learning Procedure of the Proposed Framework**Input:** Dataset (ꭙ_1_, ꭙ_2,_ ꭙ_3_), (ƴ_1_, ƴ_2_, ƴ_3_)**Output:** Trained model Ɯ, ∫1. Initialize projector Ɯ and predictor ∫;2. **repeat**3. Sample ϰ_1__→__Ƭ_, ꭚ_1__→__Ƭ_ from ꭙ_1_, ƴ_1_, respectively;4. Θ_Ɯ__←_Θ_Ɯ_ -∂/∂ Θ_Ɯ_ (ψ);5. Θ∫_←_Θ∫ -∂/∂ Θ∫ (ψ);6. **Until** Θ_Ɯ_, Θ∫ converge7. **return** Ɯ, ∫
**Algorithm 2** Procedure for Energy Consumption Prediction**Input:** ϰ_1__→__Ƭ_ State with energy consumption**Output:** ꭚ_1__→__Ƭ_1. **for** i = 1, … Ƭ do2. £_i_^Þ^ _←_ Ɯ (ϰ_i_, £_i−1_^Þ^); Computed state with total minutes3. **end for**4. ζ_1_^d^_←_ ∫d (ꭚ_0_, £_i_^Þ^);5. ꭚ_1__←_ Ʊ_ꭚ_ * ζ_1_^d^ + b_ꭚ_;6. **for** i = 2,…, Ƭ do7. ζ_1_^d^_←_ ∫ (ꭚ_i−1_, ζ_i−1_^d^);8. ꭚ_I __←_Ʊ_ꭚ_ * ζ_1_^d^ + b_ꭚ_;9. **end for**10. **return** ꭚ_1__→__Ƭ_

### 3.1. Data Collection and Pre-Processing

Currently, statistical energy data are recorded through smart meters that contain various parameters including time, date, and voltage among others. In each apartment of residential/commercial buildings, smart meters are installed that act as a center hub, where different electronic appliances and heavy machineries are fixed via wires. Normally, the meter leader collected the data from smart meters monthly, which contain several errors such as outliers, redundancy, and missing values, among others, that were mainly triggered due to faults in unit measuring tools, continuous changes in climate, consumers’ behavior, and metering glitches. Therefore, to obtain better refinement and desire for energy consumption output, it is necessary to pass these noisy data from the pre-processing step for refinement.

**Figure 3:**
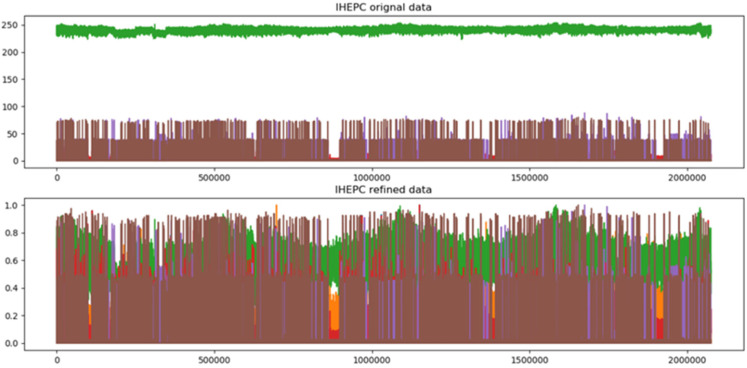
Original and refine data representation of the IHEPC dataset.

In the proposed framework, we employ a pre-processing method to refine the data before training the model because on scatter data the neural networks are more sensitive. First, we eliminate the missing values that frequently occurred during collection. Secondly, before applying standardization process, the outlier is eliminating that can affect the normalize values and drag the attribute values towards the maximum and minimum range. After normalization, the alteration effect on the IHEPC dataset is visualized in Figure 3, where the IHEPC dataset features range lies between 0 and 250. So, through assistance of min-max normalization, the diverse features range is transformed into 0 and 1.

On the other hand, a standard transformation method is also applied on KAB and solar datasets to arrange all the actual input values in a specific range. The mathematical representation of min-max and standard transform operation is shown in Equations (1) and (2).

(1) $$X=\frac{\left(Y-U\right)}{S}$$

(2) $$X=\frac{\left(Y-Y_{\min}\right)}{Y_{\max}-Y_{\min}}$$

where Y, U, S, Y_min_, and Y_max_ signify the original data, mean, standard deviation, minimum and maximum value in the dataset, respectively. Finally, we set all datasets for short intervals because we are dealing with short-term energy consumption prediction. Through assistance of pre-processing techniques on raw data we obtain remarkable prediction performance on all datasets used in this study.

### 3.2. ACL for Spatial Features Extraction

**Figure 4:**
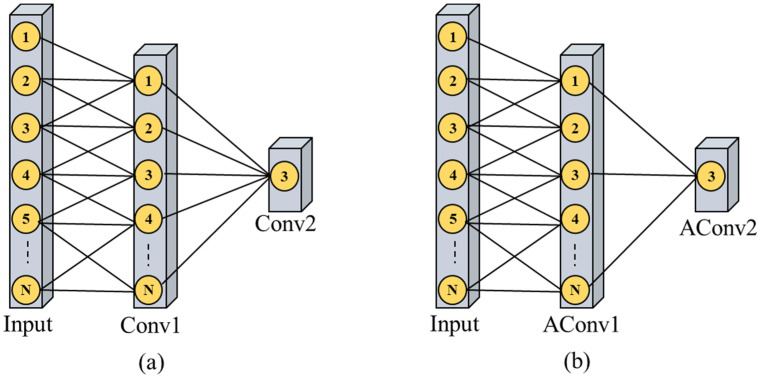
(**a**) Shows the traditional convolution layers while ACL is represented in (**b**).

CNN models are specifically developed for image analytics problems, where it takes two-dimension data as an input [45]. Recently, CNNs have shown better performance on statistical data [46]. Nowadays, one dimension CNNs have also proven remarkable performance, particularly for time series data analysis such as electricity consumption [47], weather, and stock price prediction which is a non-linear problem that mainly obtains incredible performance by utilizing the weight sharing concept. However, during the training process, CNN models extract deep features from input data by using huge numbers of parameters and loss of contextual information. To address these concerns, researchers followed various strategies including unsampling or deconvolution that require huge space in the memory and are computationally expensive. Therefore, in the proposed framework we apply ACL with the skip connections concept that is much better than the conventional CNN in terms of accuracy. The visual difference between the traditional convolution layers and ACL is shown in Figure 4.

**Figure 5:**
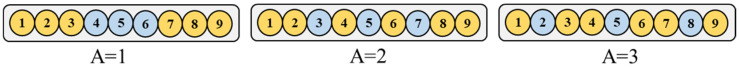
Visual representation of 1D convolution with numerous atrous rates.

Initially, atrous convolutions were design for wavelet transformation, and which expand the receptive field without an increase in parameters and are static in the feature maps size throughout the architecture. Typically, signal processing, weather forecasting, and electrical energy consumption prediction data contain lengthy patterns of information so, to extract spatial features, ACL is the best choice for the precise prediction of various types of data. Mathematically, ACL can be represented as:

(3) $$F\left(Ṩ\right)=\left(\varkappa \cdot {đ}_{f}\right)\left(Ṩ\right)=\sum_{i=0}^{k-1}f\left(i\right)\cdot \varkappa_{Ṩ-đ.i}$$

where different atrous factors, filter size, and actual input data are denoted by d, k, ϰ. For instance, we have a diverse numbers of filters f: {0, …, k − 1} → N with atrous rate 1, 2, and 3 then 1D ACL are shown in Figure 5.

### 3.3. Sequential Model for Short-Term Energy Prediction

**Figure 6:**
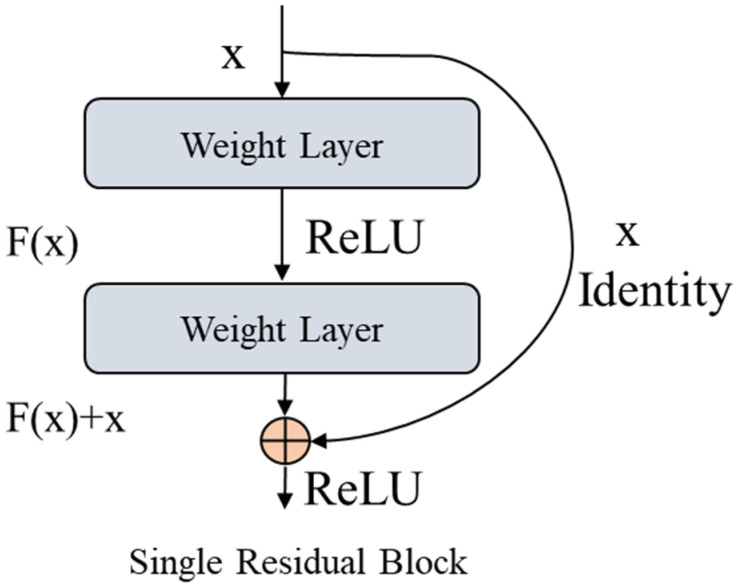
Representation of skip connection or residual concept in GRU.

In this section, we briefly explain the architecture of RGRU use in the proposed framework. In conventional neural networks, the output of one layer is passed to the next layer as an input while in the residual each layer datum is fed into the subsequent layer and the input data is forward directly to those layers that are away from two to three layers. Before a deep description below, Figure 6 shows a single residual block.

Normally, the performance of the neural network is enhanced with the increasing numbers of layers, but there is a boundary to adding the number of layers for the improvement of the accuracy. Neural networks utilized a universal function that was able to learn and evaluate any simplex or complex problem. However, sometimes it comprises a vanishing gradients problem and is not able to learn simple identity functions, even if we made complex deep architecture by increasing the number of layers in the network for improvement. Another is a degradation issue which was also created by adding the numbers of layers, where the accuracy of the network improves up to specific points and after that the performance is decreased. To overcome the overfitting and degradation problems, in this study we utilize residual or skip connection row/column wise data.

In RGRU, few layers are skipped during the training process that assists in learning an identity function directly. To understand the concept of residual block mathematically, let us assume a neural network block with x input and real distribution, represented by H(x) as shown in Equation (4).

(4) R(x)=output−input=H(x)−x

(5) H(x)=R(x)+x

Equation (5) is obtained after reordering Equation (4). The entire residual block is trying to generate true output H(x) and from Figure 6 it is clearly exhibited that x is the identity connection where the layers are truly trying to learn the residual R(x). In short, the traditional network layers are always learning from actual output H(x) while residual network layers learn from the residual R(x). It is experimentally observed that the performance of the residual is better as compared to traditional GRU because it learns from both input and output rather than only the input. Our RGRU network is capable of learning identity function and addressing the vanishing gradient problem due to the utilization of larger gradients with an initial layer that intelligently learn as fast as the resultant layers.

## 4. Experimental Results

This section is mainly classified into three steps, first a brief description about datasets, secondly three evaluation metrics are discussed in detail which are mainly used for assessment purposes, and finally experiments are conducted over KAB IHEPC, and solar datasets for short-term prediction and are compared with other existing energy consumption and generation prediction models. Further, all the experiments are conducted on GeForce GTX 2060 GPU coupled with quad channel 64 GB RAM. The model is implemented in python (Version 1.12) using Keras framework with backend TensorFlow and Adam is selected as an optimizer.

### 4.1. Dataset Description

In this study, KCB, IHEPC and solar datasets are used for experiments: the KCB energy consumption data is mainly collected from commercial buildings, while data of PV are downloaded from the Republic of Korea website. The data are recorded from January 2015 to October 2018 where the variables including surface temperature, inclined irradiance, and surrounding temperature are considered as an input while predicted variables show the output power of the plant. The details of the parameters along its numerical values are given in Table 1.

IHEPC is the residential buildings data that are publicly available on the UCI repository. These residential buildings are situated in the country of France where one-minute energy consumption data have been recorded from 2006 to 2010. In total, 2,075,259 pieces of data are collected, in which 25,979 samples contain missing values therefore, first passed it to the pre-processing layer to obtain free noisy data. It contains numerous attributes/features including data, time voltage, global active power, global reactive power, intensity, and three sub-metering as shown in Table 2. All the sub-metering data indicate the entire energy consumption in building, while sub-metering 1–3 individually record the kitchen, living and laundry room. On other hand, each sample contains 15 min of data collected from the sensor in the KCB dataset that is almost similar to IHEPC, but there are some variations in terms of time, buildings, and the recorded unit’s device.

### 4.2. Evaluation Metrics

The performance of the proposed framework is assessed through three popular evaluation metrics such as MSE, RMSE, and MAE. Equations (6)–(8) exhibit the mathematical representation of these metrics individually. MSE computes the average squared variation among the actual values and predicted output values generated by the model. RMSE is mostly used for regression model analysis that determines the dissimilarity between all the actual and predicted data, then evaluates the squares of errors and at the end computes the square root of the obtained mean value. MAE takes the average absolute of error attained through actual and model predicted values.

(6) $$\mathrm{MSE}=\frac{1}{\mathrm{Tsamp}}\sum_{1}^{n}\left(A-P\right){}^{2}$$

(7) $$\mathrm{RMSE}=\sqrt{\frac{1}{\mathrm{Tsamp}}\sum_{1}^{n}\left(A-P\right){}^{2}}$$

(8) $$\mathrm{MAE}=\frac{1}{\mathrm{Tsamp}}\sum_{1}^{n}\left|A-P\right|$$

### 4.3. Ablation Study

In this section, numerous models’ performance is evaluated over different datasets. The proposed model is built up by integrating ACL with RGRU, so to find the strength of each component, we conducted comprehensive experiments. The details of the experiments are mentioned in the following subsections.

*A*. *Evaluation over IHEPC Dataset*

**Figure 7:**
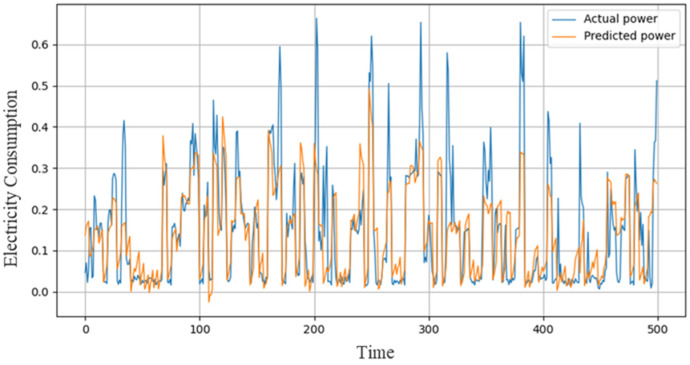
Performance of the proposed model over IHEPC dataset.

For short-term energy consumption prediction, first the ACL model is computed using IHEPC where we obtained 0.2112, 0.4595, and 0.3143 of MSE, RMSE, and MAE. Next, sequential learning model performance is checked against the same data where we achieved better results as compared to the first model, i.e., 0.1841 MSE, 0.4290 RMSE, and 0.2984 MAE. Finally, a hybrid model is proposed by incorporating ACL with sequential learning model RGRU which shows convincing performance and obtained the lowest error rate including 0.1753, 0.4186, and 0.2635 for MSE, RMSE, and MAE, respectively as shown in Table 3. The actual and predicted outcomes for the household consumption dataset on hourly resolution is illustrated in Figure 7.

*B*. *Evaluation over KCB Dataset*

**Figure 8:**
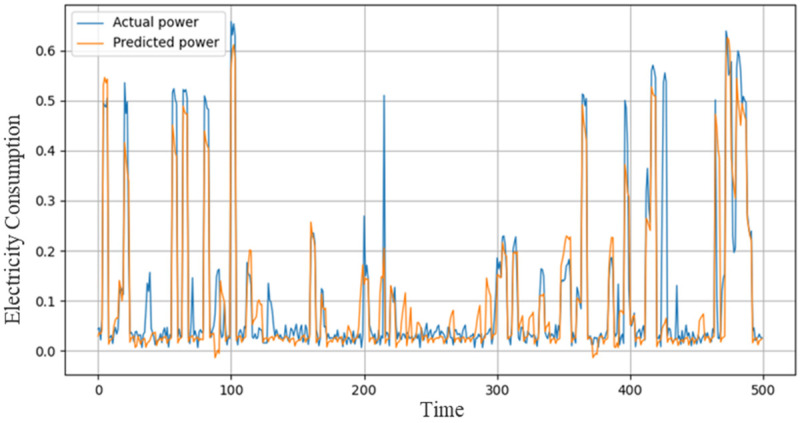
Performance of the proposed model over the KCB dataset.

On hourly resolution data of KCB, we investigated different models including ACL, RGRU, and concatenation of these two components for energy consumption prediction. This dataset is approximately similar to IHEPC where our first proposed model is ACL which obtained 0.0124, 0.1113, and 0.0928 of MSE, RMSE, and MAE. Our second proposed model is RGRU which used the concept of skip connection and performed better as compared to the first CNN model where MSE, RMSE, and MAE are 0.0121, 0.1100, and 0.0827, respectively, as shown in Table 4. Our final hybrid model shows tremendous performance and predicted next hour energy data with the smallest error score including 0.0001 MSE, 0.0100 RMSE, and 0.0031 MAE. The actual and predicted graph generated through the proposed method is shown in Figure 8.

*C*. *Evaluation over Solar Dataset*

**Figure 9:**
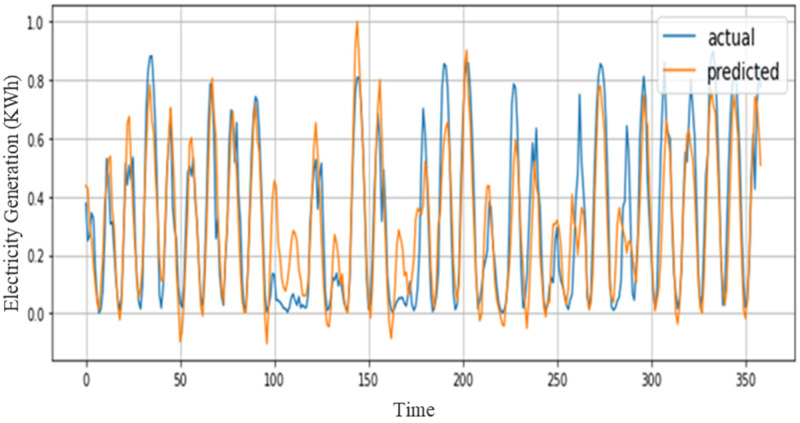
Performance of the proposed model over the solar dataset.

Nowadays, for energy management system demand-supply matching is a significant research domain. Most of the literature studies mainly focus on consumer side energy prediction which is not an efficient method for management systems at the supplier side. Regarding this along with energy consumption prediction, our proposed model also works efficiently for power generation prediction. For this job, a comprehensive result is generated over the solar dataset. Based on these energy data our first model is trained and tested, known as ACL, which obtained 0.0193 MSE, 0.1389 RMSE, and 0.1297 MAP on hourly resolution data. Next, the sequential learning model is investigated, which utilizes the residual concept known as RGRU that further boosts the performance and gives minimum error scores for MSE, RMSE, and MAE (i.e., 0.0187, 0.1367, and 0.1175) as shown in Table 5. Despite these models we also used a hybrid approach by integrating both models which also shows incredible performance such as 0.177 MSE, 0.1332 RMSE, and 0.1013 MAE. The actual and predicted graph is generated over solar data as shown in Figure 9.

### 4.4. Comparative Analysis and Discussion

In this section, the results attained via the proposed method are compared with recent techniques of energy consumption and generation prediction that also utilize the same dataset. Further, through a detailed discussion we analyze why our approach is better than the existing work.

For precise energy consumption prediction, several researchers have proposed different models. For instance, Mocanu et al. [48] investigated a novel prediction model to improve the infrastructure of the electricity and efficient implementation of renewable energy sources, however their model generated a high error score because they extracted short descriptors which did not entirely cover the meaningful information from the input data. Another approach introduced by Marino et al. [49] aims to reduce energy from wastage and predict energy with minimum error. This work is further boosted by Ullah et al. [32] who utilized CNN with Bi-directional BDLSTM and obtained 0.31 MSE, 0.56 RMSE, and 0.34 MAE. In traditional convolution layers, where various filters are applied that cover a small region of input data, the generated feature maps do not contain significant information. Similarly, Le et al. [50] also practice the CNN with Bi-LSTM for the efficient prediction of consumed energy. For the same problem, Kim and Cho [51] proposed DL using an autoencoder and analyzed different resolution data to confirm the precise prediction. Autoencoders compress the input data and ignore small significant information which also plays a major role in prediction. Due to the growing human population, residential buildings consume large amount of energy, and it is common knowledge that people in residential buildings have no information on how to preserve the energy. Therefore, to stabilize the energy and establish a convenient method of communication between consumers and suppliers, Kim and Cho presented a hybrid model using CNN with LSTM for prediction which did not extract discriminative features, hence they obtained a high error rate.

From Table 6 we observed that the proposed ACL-RGRU obtained remarkable performance as compared to previous state-of-the-art approaches. To obtain better results, most of the researchers followed the concept of adding layers but, in the proposed model, we gained minimum error rate by using the concept of RGRU. On the IHEPC dataset our method attained the 0.1753, 0.4186, and 0.2635 error score for MSE, RMSE, and MAE, respectively. Another effort by Rajabi et al. [52] obtained 0.79 RMSE and 0.59 MAE through two-dimensional CNN. Another approach presented by Khan et al. [53] further boosted this work where they established a hybrid approach by integrating CNN with an autoencoder for precise energy prediction and provided efficient communication between consumers and suppliers where they obtained an 0.19, 0.47, and 0.31 error rate for MSE, RMSE, and MAE, respectively. A study followed by Sajjad et al. [54] applied pre-processing techniques before extracting patterns from data through CNN and at the end they utilized GRU for sequence learning, which further reduced the error with 0.22, 0.47, and 0.33 for MSE, RMSE, and MAE. Besides these most recent approaches, CNN with bidirectional GRU is developed by Khan et al. [21] where they further enhanced the prediction scores with 0.18, 0.42, and 0.29 for MSE, RMSE, and MAE.

We also trained and tested the proposed model over our own prepared dataset where data are basically collected from sensors attached with commercial buildings that count the energy consumption units. The attributes are entirely similar to IHEPC, but there exist some variations which are briefly discussed in the dataset description section. On KCB there are only two articles that exist in the literature, but authors in [53] only mentioned that their proposed model gives the worst performance while the LSTM model achieved the highest performance and did not mention numerical results. Another recent work by Khan et al. [57] proposed a hybrid model by the integration of dilated convolutional layers with bidirectional long-short-term-memory. On hourly data they obtained 0.0126, 0.1122, 0.0893 sores for MSE, RMSE, and MAE. Furthermore, the dataset also contains noisy and scattered data, therefore the authors first applied some pre-processing steps such as filling missing values, removing outliers and performing some normalization techniques in order to arrange all the values in a specified threshold. In contrast, utilizing the concept of ACL with RGRU block shows remarkable predicted scores of 0.0001, 0.0100, and 0.0031 for MSE, RMSE, and MAE.

We deeply investigated the literature studies and concluded that researchers only focused on energy consumption and limited work has been presented that accomplished both consumption and generation prediction in a unified framework. For better energy management it is necessary to build a model that effectively predicts the renewable energy to fulfill the demand of customers. Therefore, in this study, a generalize hybrid deep learning model is proposed for equivalent consumer power demand with supply. Our model obtained 0.0177, 0.1332, and 0.1013 scores for MSE, RMSE, and MAE. For a fair comparison we found that only one article is present which also used the same solar dataset. An adaptive artificial neural network is proposed by Zameen and Won [58] and achieved 0.0207 and 0.1438 scores for MSE and RMSE. The results clearly verify and demonstrate that the proposed hybrid approach is suitable and performs better for both energy consumption and generation prediction.

The learning mechanism of the proposed model is different from the above discussed literature due the following aspects: by using ACL broadly to cover the input data it is capable of extracting discriminative, robust, and representative features. Furthermore, our approach is also deployable in real-time because ACL reduces huge numbers of model parameters. Another big advantage of the usage of the skip connection concept that intelligently improves the network performance and reduces the chances of overfitting. Due to the above, reasons the proposed model achieved new state-of-the-art accuracy.

### 4.5. Time Complexity Analysis

We calculate and compare the time complexity of the proposed model to ensure its deployment for real-time applications. To accomplish this job, we utilize two local servers: CPU and GPU. In this study, the solar dataset is used, so total training and testing time over CPU intel (R) Core (TM) i7-7700 with 16.0 GB RAM is 640 and 0.08 s, respectively. Meanwhile the training and testing time of the proposed model with the same resolution data using GPU (GeForce RTX 2060) Super with 64 GB RAM installed Window 10 operating system and processor AMD Ryzen 9 is 328 and 0.001 (s). The model training and testing execution time is also given in Table 7.

## 5. Conclusions and Future Research Directions

In this paper, a short-term energy prediction framework is proposed that mainly contains four steps. First, the raw data is collected that contain abnormalities including missing values, outliers, and redundancy among others, therefore before training it is necessary to polish these data. Secondly, to obtain abnormality free data we passed it to pre-processing where a normalization method is applied to organize all the data into a particular range. Next, discriminative features are extracted via ACL followed by sequence learning model RGRU which learns spatial patterns during training process. Finally, the proposed framework is assessed over KCB, IHEPC, and solar datasets that shows incredible performance as compared to other state-of-the-art energy prediction techniques. In addition, three evaluation metrics are used including MSE, RMSE, and MAE for the assessment of the proposed framework. The limitation of this work is that the proposed model is only designed for multi-step prediction with a particular horizon. To adopt such limitations in future, we will explore some other approaches such as spiking neural network, fuzzy logic, and lightweight hybrid model, particularly for single-step forecasting and resource constraining devices.

## Figures and Tables

**Table 1:** Detailed description of the solar dataset with parameters and units.

#	Parameters	Values
01	Plant Maximum Output	2610 kW
02	Plant Capacity	3026 kW
03	Maximum Inclined Irradiance	999.96
04	Maximum Surface Temperature	49.78
05	Maximum Surrounding Temperature	125.60
06	Duration	(2015–2018)
07	Time Internal	1 h
08	Data Points	17,252

**Table 2:** Detailed description of the IHEPC residential dataset with features and units.

#	Available Features	Units	Detail Description
01	Date	dd/mm/yy	All data contain integer values in proper range and format (1–30), (1–20), (2006–2010) days/months/years
02	Time	hh/mm/ss	All the time is given in hours/minutes/seconds
03	Global Active Power	kiloWatts (kW)	1 min’s data are recorded
04	Global Reactive Power		
05	Voltage	Volts (V)	1 min’s mean value in voltage
06	Global Intensity	Amp	1 min average for intensity
07	Sub-Metering 1	Watt-hour (Wh)	All metering data are related to kitchen appliances such as oven, microwave. Living house-associated appliances such as washing machine, freezer. Bedroom involves air conditioners and heaters.
08	Sub-Metering 2		
09	Sub-Metering 3		

**Table 3:** Various models performance over the IHEPC dataset using hourly resolution data. Bolds results demonstrate the best performance.

Method	MSE	RMSE	MAE
ACL	0.2112	0.4595	0.3143
RGRU	0.1841	0.4290	0.2984
**Proposed**	**0.1753**	**0.4186**	**0.2635**

**Table 4:** Various models performance over the KAB dataset using hourly resolution data. Bold results demonstrate the best performance.

Method	MSE	RMSE	MAE
ACL	0.0124	0.1113	0.0928
RGRU	0.0121	0.1100	0.0827
**Proposed**	**0.0001**	**0.0100**	**0.0031**

**Table 5:** Various models performance over the solar dataset using hourly resolution data. Bold results demonstrate the best performance.

Method	MSE	RMSE	MAE
ACL	0.0193	0.1389	0.1297
RGRU	0.0187	0.1367	0.1175
**Proposed**	**0.0177**	**0.1332**	**0.1013**

**Table 6:** Comparative analysis of the proposed method with state-of-the-art for hourly data resolution using IHEPC. Bold and underlined results demonstrate the best and runner-up performance.

References	Method	MSE	RMSE	MAE
Mocanu et al. [48] 2016	FCRBM	-	0.66	-
Marino et al. [49] 2016	LSTM-S2S	0.55	0.74	-
Ullah et al. [32] 2019	MLDB-BLSTM	0.31	0.56	0.34
Le et al. [50] 2019	CNN-BiLSTM	0.29	0.54	0.39
Rajabi et al. [52] 2019	CNN-2D-RP	-	0.79	0.59
Kim and Cho [51] 2019	SE-AE	0.38	-	0.39
Kim and Cho [55] 2019	CNN-LSTM	0.37	0.61	0.34
Khan et al. [53] 2020	CNN+LSTM-AE	0.19	0.47	0.31
Sajjad et al. [54] 2020	CNN-GRU	0.22	0.47	0.33
Khan et al. [21] 2020	CNN-MB-GRU	0.18	0.42	0.29
Jun and Cho [56] 2020	CNN-LSTM-MHA	0.26	-	-
Khan et al. [57] 2021	AB	0.56	0.75	0.67
**Proposed**	**ACL+RGRU**	**0.17**	**0.41**	**0.26**

**Table 7:** Execution time of the proposed model using the solar dataset.

Local Systems	Training Time (s)	Testing Time (s)
CPU	640	0.08
GPU	328	0.001

## Data Availability

Not applicable.
